# Lubricin binds cartilage proteins, cartilage oligomeric matrix protein, fibronectin and collagen II at the cartilage surface

**DOI:** 10.1038/s41598-017-13558-y

**Published:** 2017-10-13

**Authors:** Sarah A. Flowers, Agata Zieba, Jessica Örnros, Chunsheng Jin, Ola Rolfson, Lena I. Björkman, Thomas Eisler, Sebastian Kalamajski, Masood Kamali-Moghaddam, Niclas G. Karlsson

**Affiliations:** 1 Department of Medical Biochemistry and Cell Biology, Institute of Biomedicine, Sahlgrenska Academy, University of Gothenburg, Gothenburg, Sweden; 2 Department of Immunology, Genetics and Pathology, Science for Life Laboratory, Uppsala University, Uppsala, Sweden; 3 Department of Orthopaedics, Institute of Clinical Sciences, The Sahlgrenska Academy, University of Gothenburg, Gothenburg, Sweden; 4 Department of Rheumatology and Inflammation Research, Institute of Medicine, Sahlgrenska Academy, University of Gothenburg, Gothenburg, Sweden; 5 Department of Clinical Sciences, Danderyd Hospital, Karolinska Institutet, Stockholm, Sweden; 6 Department of Molecular Skeletal Biology, Lund University, Lund, Sweden

## Abstract

Lubricin, a heavily *O*-glycosylated protein, is essential for boundary lubrication of articular cartilage. Strong surface adherence of lubricin is required given the extreme force it must withstand. Disulfide bound complexes of lubricin and cartilage oligomeric matrix protein (COMP) have recently been identified in arthritic synovial fluid suggesting they may be lost from the cartilage surface in osteoarthritis and inflammatory arthritis. This investigation was undertaken to localise COMP-lubricin complexes within cartilage and investigate if other cartilage proteins are involved in anchoring lubricin to the joint. Immunohistochemical analysis of human cartilage biopsies showed lubricin and COMP co-localise to the cartilage surface. COMP knockout mice, however, presented with a lubricin layer on the articular cartilage leading to the further investigation of additional lubricin binding mechanisms. Proximity ligation assays (PLA) on human cartilage biopsies was used to localise additional lubricin binding partners and demonstrated that lubricin bound COMP, but also fibronectin and collagen II on the cartilage surface. Fibronectin and collagen II binding to lubricin was confirmed and characterised by solid phase binding assays with recombinant lubricin fragments. Overall, COMP, fibronectin and collagen II bind lubricin, exposed on the articular cartilage surface suggesting they may be involved in maintaining essential boundary lubrication.

## Introduction

Arthritis is a large heterogeneous group of highly prevalent erosive joint disorders with the two most common being osteoarthritis (OA) and rheumatoid arthritis (RA). OA begins with pathological mechanical stress to the joint cartilage, and risk factors include trauma, increasing age and hereditary factors^1^. This initial damage goes on to affect inflammatory cytokines resulting in an imbalance in cartilage catabolism creating a more complex disease^2^. RA is a systemic chronic autoimmune inflammatory disease with joint surface destruction due to the destructive inflammatory and multifaceted immune response^3^. Arthritic diseases are characterised by pain and loss of range of motion, often leading to severe disability and increased health costs. While the treatments for the source of joint damage for RA continue to improve^4^, this is not the case for the more common OA, affecting 10% of men and 13% of women aged 60 years or older in the US, where disease management is central until a time when surgery is unavoidable for severely affected patients^5^.

Maintenance of the superficial cartilage surface of diarthrodial joints is essential for the lubricating and shock-absorbing properties of the joint. The preservation or re-establishment of the joint surface is essential for improving the joint function and mobility of arthritis patients, particularly in OA, where current treatments are limited^1–4^. Effective boundary lubrication, found at the surface of the cartilage, is highly reliant on the heavily *O*-glycosylated protein lubricin, which is synthesised by synoviocytes and articular chondrocytes and abundant in the synovial fluid (SF), synovial membrane and superficial zone of articular cartilage^6–9^. Lubricin holds predominantly core 1 *O*-glycan structures with sialic acid giving the protein a negatively charged central domain capped by positively charged termini^10,11^. This glycan component likely contributes to the protein’s low friction and non-adherent lubricating properties^12^. The extreme load and shear forces that the cartilage surface withstands means that the surface lubricin layer must adhere strongly to the cartilage surface^13,14^.

Articular cartilage, found in synovial joints such as the knee, hip and shoulder, functions foremost for load-supporting and load transferring between bones^15^. It is an avascular tissue with minimal cellularity resulting in a low proliferative environment making restoring arthritic damage difficult^15^. Articular cartilage is composed predominately of water, up to 70–80%, with the matrix forming component of the extracellular matrix (ECM) including a range of often highly post translationally modified proteins including collagens, proteoglycans such as aggrecan, laminins and fibronectin, reviewed in detail elsewhere^15,16^.

Collagen II is the most abundant collagen in articular cartilage and forms extended fibrils creating a network with other proteins including other collagens and proteoglycans^17,18^. Another fibril forming protein important for matrix formation is the glycoprotein fibronectin, which consists of two disulfide bound subunits of approximately 250 kDa^19^. Fibronectin aids ECM formation by interacting with a range of ECM components including collagen, where it may organise the ECM network^20^. COMP, a homopentameric glycoprotein of the thrombospondin family consisting of 100–110 kDa subunits synthesised by chondrocytes^21^, is of lower abundance in cartilage, however, its complex forming capabilities remain pivotal^22–24^. The globular C-terminal region of COMP is important for binding to aggrecan^22^, fibronectin^23^ and collagen I, II and IX^24–27^, as well as forming non-covalent bonds with lubricin. COMP also forms covalent, disulfide bonds with lubricin, however, it is the N-terminal region (aa 102–191 and 287–504) involved in forming these bonds^28^. Lubricin has been shown to bind to other cartilage components including fibronectin in surface force apparatus experiments^29^, and to galectin-3 at the cartilage surface^30^. Lubricin, fibronectin and collagen II have been identified co-localised at the surface of engineered meniscal tissue^31^.

Our previous analysis has shown specific disulfide bonds and non-covalent interactions that complex lubricin, a protein of the cartilage and SF, with the cartilage protein COMP^28^. These complexes were identified in the SF of arthritic patients, suggesting that the complexes could have been lost from the articular cartilage surface as part of the disease process. Here, the nature and location of COMP-lubricin complex in the cartilage tissue was investigated to determine if this bond adheres lubricin to the cartilage surface. Analysis was also performed to identify other cartilage binding partners that may also be involved in the adherence of lubricin to the cartilage surface.

## Results

### Immunohistochemical co-localisation of lubricin and COMP on the cartilage surface

**Figure 1:**
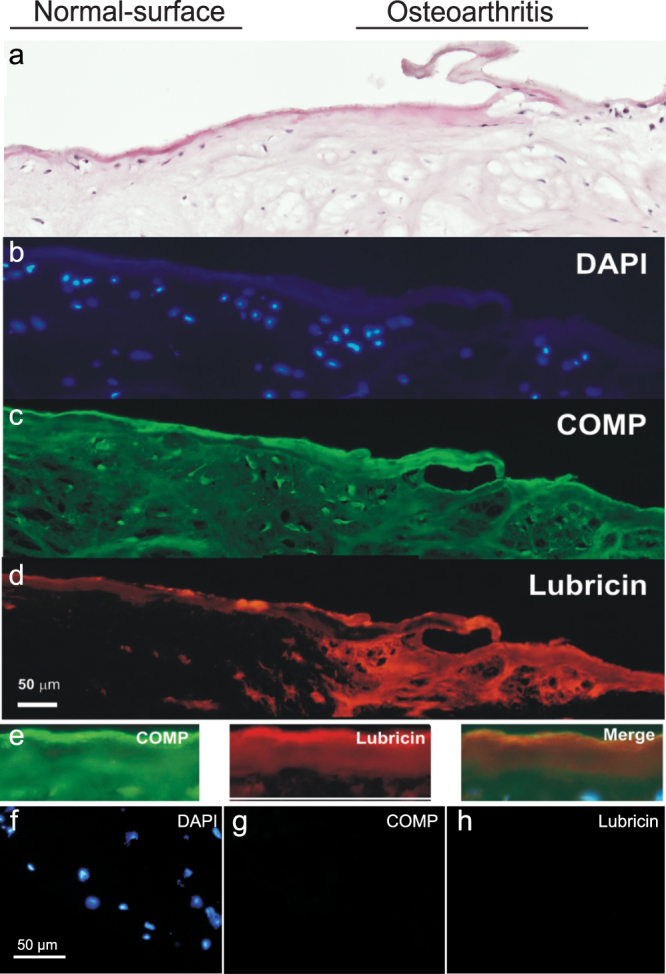
Immunohistochemical co-localisation of lubricin and COMP. Dual antibody immunofluorescence on OA cartilage biopsy cryosections with anti-lubricin (P3-118) and anti-COMP (mAb 16F12) antibodies. Section shows an area of undisrupted cartilage surface (left side) and an area more severely affected by OA including a tear (right side). (**a**) Haemotoxylin and esosin staining. (**b**) DAPI staining for nuclei of chondrocytes. (**c**) COMP (green) was constantly distributed over the section with greater intensity on the superficial zone. (**d**) Lubricin (red) was present on the superficial zone of the cartilage and into the superficial zone in the area of the tissue with OA degradation at the surface. (**e**) Cartilage surface alone including COMP antibody staining, lubricin antibody staining and merged image showing co-localisation of COMP and lubricin on cartilage surface. Negative controls of dual antibody immunofluorescence analysis. The specificity of the staining was verified using matched isotype negative controls or control serum at the same concentration as the primary antibodies. (**f**) Negative control showing DAPI staining. (**g**) Negative control for the anti-COMP antibody. (**h**) Negative control for the anti-lubricin antibody.

The co-localisation of COMP and lubricin was investigated by immunohistochemistry (Fig. 1). Human cartilage biopsies including the subchondral bone were obtained from non-weight bearing ventral areas of the femoral trochlea peripheral to the femoropatellar joint during medial unicompartmental knee arthroplasty surgery. Both patients, (n = 2, both male aged 67 and 79 years of age at surgery) had primary, anterior idiopathic OA and intact cruciate ligaments. All biopsies were acquired from the border between macroscopically normal appearing cartilage and areas with obvious cartilage degradation. Matched isotype and normal serum negative controls confirmed staining specificity (Fig. 1f–h).

The representative tissue section in Fig. 1 contains an area of smooth, relatively normal cartilage surface (left side) and an adjoining distorted, highly OA affected area including disrupted cartilage with tear and cartilage flap (right side). There is a reduction in cells in the superficial zone and oedema, particularly under the disrupted cartilage surface. COMP (Fig. 1c and e) was distributed throughout the interstitial matrix with stronger staining on the superficial layer; approximately 10 µm deep in the healthy tissue, up to 30 µm in the OA affected area. Lubricin (Fig. 1d and e) staining in the less severely OA affected tissue (left hand side of slide) was strong on the very edge of the cartilage with lesser staining up to 25 µm in depth. In the OA affected area, strong lubricin staining was present up to 150 µm into the tissue. This shows that in more normal appearing cartilage surface tissue, lubricin and COMP are co-localised only on the superficial surface of the cartilage. In OA affected tissue, lubricin production appeared to be upregulated, possibly to re-establish a functional lubricative layer.

### The distribution of Lubricin on the cartilage surface of COMP Knockout (KO) mice

**Figure 2:**
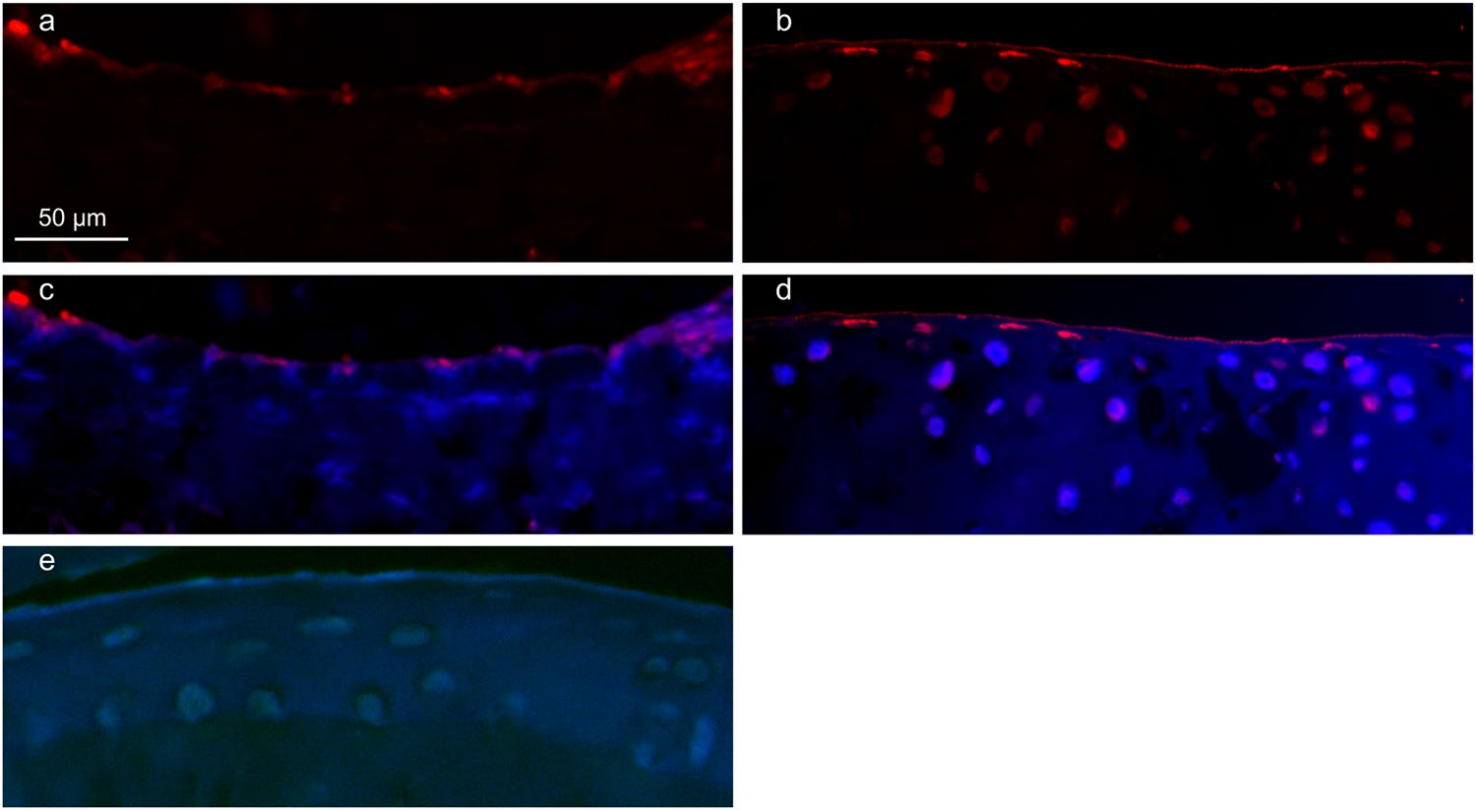
Immunohistochemical localisation of lubricin in WT and COMP KO mice paw joint cartilage tissue. (**a**) WT mouse tissue stained with rabbit anti-lubricin (P3-118) visualised with Rhodamine Red-X shows a diffuse lubricin layer at the cartilage surface. (**b**) COMP KO mouse tissue stained with rabbit anti-lubricin (P3-118) visualised with Rhodamine Red-X showing a discrete, distinct layer of lubricin at the cartilage surface. (**c**) WT mouse tissue stained with rabbit anti-lubricin (P3-118) visualised with Rhodamine Red-X also showing DAPI staining. (**d**) COMP KO mouse tissue stained with rabbit anti-lubricin (P3-118) visualised with Rhodamine Red-X also showing DAPI staining. (**e**) Negative control of WT mouse paw tissue performed by omitting primary antibody.

Given that COMP KO mice do not show massive deformity compared to wildtype (WT) animals^32^, and it is known that lubricin is essential for boundary lubrication, immunohistochemistry (IHC) was used to evaluate the presence of lubricin on the cartilage of COMP KO mice. WT mice showed lubricin staining with the most intense staining in the superficial layer chondrocytes with little staining of the cartilage surface (Fig. 2a and c). COMP KO tissue also stained with lubricin with clear staining in chondrocytes deeper into the tissue than observed in WT tissue as well as more lubricin apparent on the cartilage surface illustrated by the sharper staining at the surface edge (Fig. 2b and d). Negative control is shown in Fig. 2e.

Although lubricin appeared more intense in COMP KO mice, it should be noted that the cartilage tissue in the COMP KO mouse seemed generally less dense as observed by the dense ECM obscuring the DAPI-stained nuclei in the WT tissue compared to the clear nuclei in the KO tissue (Fig. 2c and d). This reduced density in KO mice could have allowed more efficient lubricin staining. It is clear, however, that lubricin was present on the cartilage surface in both WT and COMP KO mice, suggesting interactions other than COMP may be important in the adherence of lubricin to the cartilage surface in mice.

### Proximity ligation assay (PLA) of lubricin and possible binding partners

**Figure 3:**
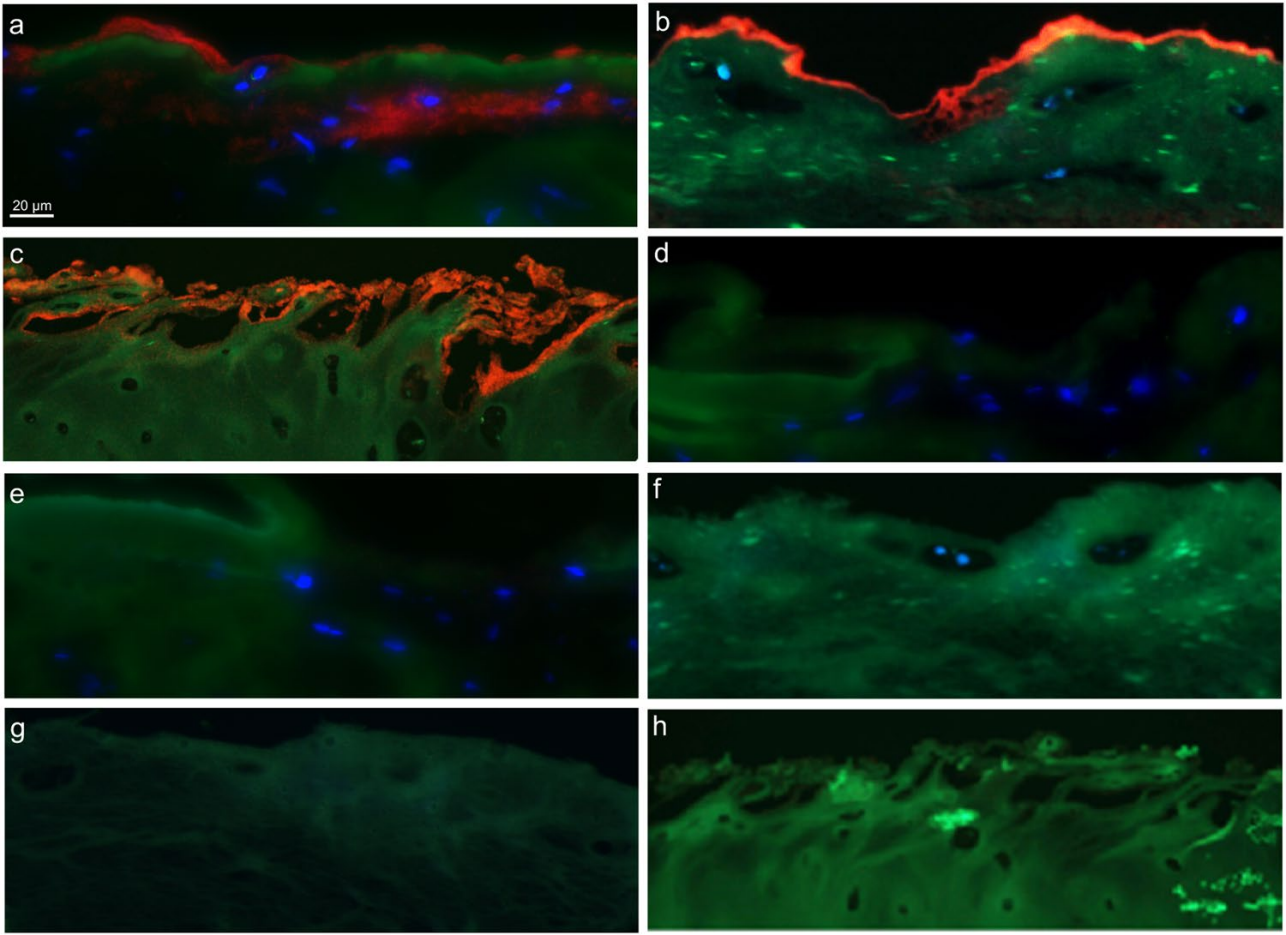
*In situ* PLA of lubricin with possible cartilage binding partners. *In situ* PLA was performed on OA cartilage biopsy cryosections with the following pairs. *In situ* PLA uses two primary antibodies targeting proteins of interest and secondary antibodies conjugated to DNA oligonucleotides (PLA probes). If the two proteins of interest are in a complex the two PLA probes will be in close enough proximity to bind and facilitate formation of a circular DNA molecule using two additional DNA oligonucleotides which then undergo rolling circle amplification to enhance the signal. Fluorescently (red) labelled complementary DNA is then added in order to visualise the DNA. (**a**) Lubricin (PA3-118) and COMP (16F12). Bound lubricin and COMP (red) were identified at the cartilage surface. A second layer was also apparent below the surface. (**b**) Lubricin (5C11) and fibronectin (ab32419). Bound lubricin and fibronectin (red) were predominately identified at the cartilage surface. Only when the PLA signal was thinner at the surface was signal observed just below the surface. (**c**) Lubricin (5C11) and collagen II (sc-7763). Bound lubricin and collagen II (red) were identified only at the surface with no PLA signal further into the tissue. PLA signal are red, DAPI staining in blue. For the 3 antibody pairs, 5 separate antibodies were used as anti-lubricin 5CII was used for 2 pairs. Negative controls were performed by omitting each primary antibody for all of the five antibodies used. (**d**) lubricin negative control (PA3-118), (**e**) COMP negative control (16F12), (**f**) lubricin negative control (5CII), (**g**) fibronectin negative control (ab32419) and (**h**) collagen II negative control (sc-7763).

Given this indication that other binding partners may be involved in lubricin adherence to the cartilage surface, two proteins important in cartilage ECM formation, fibronectin and collagen II, were investigated in human tissue sections. PLA was used to directly identify protein complexes formed between lubricin and other likely cartilage binding proteins fibronectin and collagen II, as well as COMP, *in situ* ^33^. Negative controls are shown in Fig. 3d–h.

As expected the PLA of lubricin and COMP (Fig. 3a) confirmed an interaction between the two proteins at the cartilage surface. Interestingly, the PLA signal from the COMP-lubricin complex was strong up to 40 µm into the cartilage, particularly intense where the surface layer was diminished. PLA signals were also detected for both the lubricin and fibronectin (Fig. 3b) and lubricin and collagen II (Fig. 3c) pairs. The latter two pairs were only identified at the cartilage surface with no protein interactions apparent deeper in the tissue. This suggests that both fibronectin and collagen II, along with COMP may be involved in the adherence of lubricin to the cartilage surface.

### Solid phase binding assays

**Figure 4:**
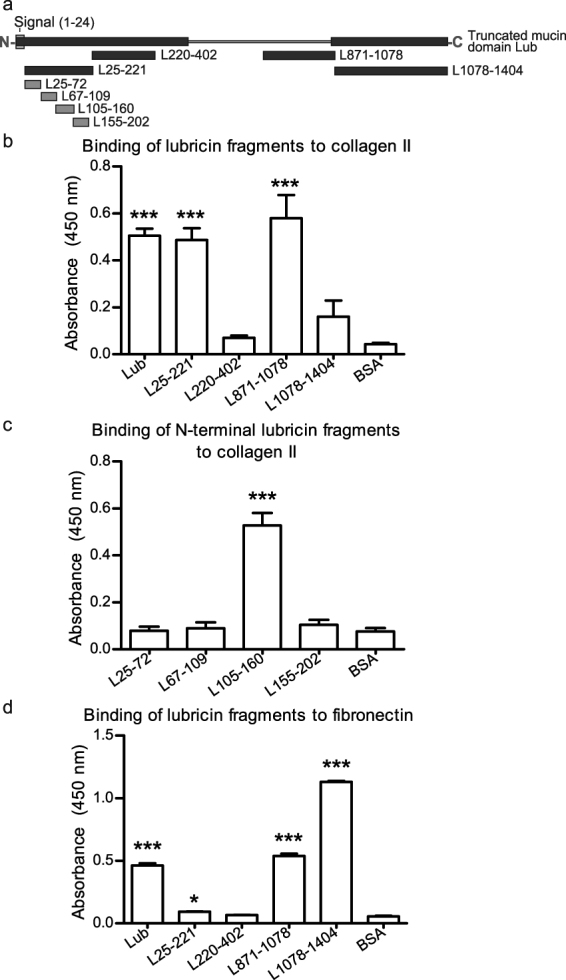
Recombinant (RC) lubricin forms non-covalent complexes with collagen II and fibronectin. (**a**) Representation of RC lubricin constructs. Dark blue: FLAG-tagged, expressed in mammalian 293 F cells. Light blue: N-terminal fragments GST-tagged expressed in *E. coli* strain Rosetta 2. (**b**) Interaction between collagen II isolated from bovine cartilage and RC lubricin fragments, full length lubricin and BSA by solid phase binding assay. (**c**) Interaction between collagen II isolated from bovine cartilage and RC N-terminal lubricin fragments and BSA by solid phase binding assay. (**d**) Interaction between fibronectin isolated from human blood and RC lubricin fragments, full length lubricin and BSA by solid phase binding assay. For all assays n = 3 and error bars are standard deviation. N- designates the amino terminus and –C designates the carboxy terminus. The signal peptide (1–24) is shown in grey. All statistical analyses compare the binding of lubricin fragments to the binding of the BSA standard. * is defined as p ≤ 0.05, and *** is defined as p ≤ 0.001.

To verify that collagen II and fibronectin bind lubricin and to determine the lubricin binding region, solid phase binding assays were performed using a range of lubricin fragments that have been previously described^28^. Statistical analyses compared the binding of each recombinant lubricin fragment to bovine serum albumin (BSA) binding. Collagen II isolated from bovine cartilage bound the full length lubricin (lub) with a truncated mucin domain, the L871-1078 mucin domain construct and the N-terminal fragment L25-221 (Fig. 4b) to a similar extent. Four smaller N-terminal lubricin fragments were then tested and collagen II was found to bind only the L105-160 fragment (Fig. 4c) when compared to BSA. Human blood fibronectin bound with similar intensity to the full length-truncated mucin domain lubricin and L871-1078 mucin domain fragment; however, fibronectin binding was more than twice as intense to the C-terminal fragment L1078-1404 (Fig. 4d). Together, these results verify that lubricin binds collagen II and fibronectin and that the N-terminal of lubricin is important for binding with collagen II and the C-terminal for fibronectin.

### Identification of lubricin from digested cartilage biopsy tissue

**Figure 5:**
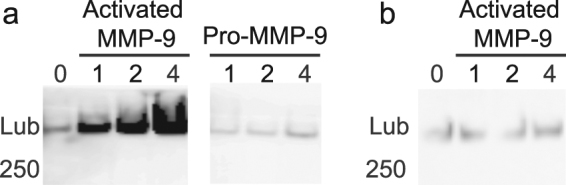
Western blots of lubricin released from the cartilage surface by MMP-9. (**a**) Cartilage biopsy tissue pieces were incubated with pro- and activated MMP-9 and released proteins were separated by SDS-PAGE and analysed by Western blot with lubricin antibody mAb13. (**b**) Purified lubricin was also incubated with activated MMP-9. Lubricin was released from the cartilage biopsy pieces when the surface of the cartilage was digested by MMP enzymes which digest the proteins of the cartilage ECM.

To confirm the observed superficial lubricin was directly at the cartilage surface, pieces of biopsy tissue were digested with matrix metalloproteinase-9 (MMP-9). Tissue was digested with activated MMP-9 as well as incubated with the pro-enzymes. MMPs effectively digest proteins of the cartilage ECM. MMP-9 (gelatinase B) cleaves elastin, aggrecan, laminin, COMP and collagens IV, V, XI, XIV^34,35^. MMP-9 released lubricin from the pieces of decalcified OA patient articular cartilage biopsies into the buffer (Fig. 5a, complete Western blot shown in supplementary Fig. S1). Purified lubricin was also incubated with activated MMP9, which demonstrated that lubricin itself is not a substrate for the enzyme (Fig. 5b, complete Western blot shown in supplementary Fig. S1), eliminating the possibility that lubricin being released from the cartilage occurred through its direct digestion.

## Discussion

Co-localisation of COMP and lubricin on the cartilage surface suggested the two are bound on the surface, an observation confirmed by *in situ* PLA. The presence of lubricin on the outer surface was confirmed by human biopsy digestion with the ECM destructive enzyme MMP-9 which released lubricin from the cartilage surface as identified by Western blot. Further PLA analyses identified that lubricin also binds fibronectin and collagen II at the cartilage surface. These data suggest a robust redundant mechanism where COMP, fibronectin and collagen II anchor lubricin to the cartilage surface to aid in the creation of the essential boundary lubrication of the joint.

The disulfide-bound COMP-lubricin complex was initially identified in arthritic SF by mass spectrometry^28^, however, the concentration of COMP is far greater in cartilage compared to SF^36^, suggesting the complex originates in the cartilage. The superficial layer is lost in OA through physical destruction or perhaps by enzymatic means such as increased MMP-3 degradation^37^. Lubricin and COMP co-localised at the superficial zone of cartilage, with lubricin expression deeper in areas of damage, indicating damaged cartilage tissue may attempt to reform the surface structure. Lubricin is expressed in the superficial zone of the cartilage in healthy tissue^6^, however, lubricin protein expression has been shown to be upregulated after mechanical injury^38^. Bovine explants showed lubricin staining deeper into the cartilage tissue in areas of injury^38^, as observed in this study in the damaged tissue. Altered COMP expression patterns in patients with arthritis have been identified^39^, and stronger expression observed in the superficial fibrillated cartilage was shown here. *In situ* PLA corroborated this result and confirmed that COMP and lubricin are bound at the cartilage surface. A large accumulation of COMP-lubricin complexes identified deeper into the cartilage tissue suggests that the complex may be produced in the cartilage as a precursor reservoir, and migrate through the tissue to be held on the cartilage surface providing a renewable source of the lubricin layer.

Given the necessity of the lubricin layer in maintaining joint articulation, it is possible that a redundant mechanism is involved. This is evidenced by the finding that COMP KO mice have normal development and histology of the skeleton^32^. Investigation of the superficial lubricin layer in COMP KO mice in our study demonstrated that the mice retain lubricin on articular cartilage. The lubricin layer even appeared more intense than in wildtype mice, although this is likely due to the loss of cartilage tissue density in the KO mice given COMP does not appear to be compensated for by other members of the thrombospondin family^32^. It is important to consider that the significance of COMP may be diminished in smaller animals, for instance, mice do not have COMP in their tendons^32^ unlike larger animals including humans^40^ and bovine^41^. Nevertheless, the identification of a lubricin layer on COMP KO mice articular cartilage suggests that other mechanisms may also be involved in lubricin retention in humans. Disruption to COMP folding by mutation causes devastating human diseases including multiple epiphyseal dysplasia (MED)^42^, and pseudoachondroplasia (PSACH)^43^, which can be replicated in mice^44^. PSACH causes disproportionate short stature, early onset OA and debilitating joint pain from childhood^45^, a result of the intracellular accumulation of misfolded COMP in the rough endoplasmic reticulum of chondrocytes^46,47^. This leads to the premature death of growth plate chondrocytes^48^ and it is hypothesised that this, rather than the loss of COMP itself, results in PSACH^32,43^. Overall, although it is clear that COMP and lubricin complex at the cartilage surface, the loss of COMP does not lead to complete loss of boundary lubrication, hence additional mechanisms appear to be involved in the adherence of lubricin to the cartilage surface.

The two proteins chosen for investigation as possible lubricin binding partners, fibronectin and collagen II, are both important in ECM formation, and fibronectin has been reported to co-purify during enrichment of synovial lubricin^10^. Using *in situ* PLA, we demonstrated that both proteins are capable of forming proximity complexes *in situ* with lubricin. Unlike COMP, the fibronectin-lubricin and collagen II-lubricin complexes were only identified on the outer surface of articular cartilage from OA patients. The role of fibronectin in the cartilage superficial zone is not well characterised, however, it is found throughout the cartilage tissue, increased at the articular surface^49^ and upregulated in OA^49,50^. Lubricin has been shown to tether to a fibronectin layer and this dual-protein layer provided coefficients of friction similar to that observed for lubricin alone suggesting strong binding between the two proteins resulting in effective wear protection^29^.

Collagen II is abundant throughout the articular cartilage^17^. Collagen has also been shown to interact with lubricin by quartz crystal microbalance with dissipation experiments. The lubricin bound spontaneously and could not be removed by interference with serum albumin^51^, again suggesting a strong bond resulting in a surface with a low coefficient of friction. Collagen II, along with fibronectin, was identified to co-localise with lubricin on engineered meniscal tissue, collagen gel seeded with fibrochondrocytes^31^. Here we have confirmed that both fibronectin and collagen II form complexes with lubricin *in situ* and these complexes are present at the superficial zone of articular cartilage. It has also been shown that Galectin-3, a carbohydrate-recognition-binding-domain containing protein, binds lubricin at the cartilage surface, enhancing boundary lubrication^30^. Overall, this redundant binding mechanism shows that the retention of lubricin on the cartilage surface is essential for cartilage health.

**Figure 6:**
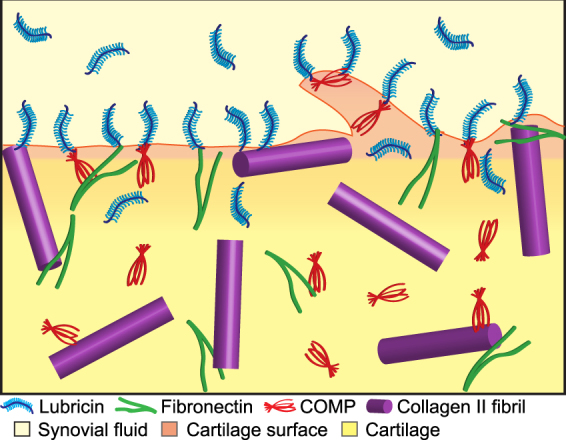
Illustration of the cartilage surface showing protein interactions. Lubricin is blue with glycosylation shown in light blue, COMP is red shown as the pentameric structure, fibronectin is green shown as the dimeric structure and collagen II is purple shown as collagen II fibrils. The SF is shown in light straw, the cartilage in yellow and the cartilage surface in pink. COMP, collagen II and fibronectin adhere lubricin, primarily by the termini of lubricin, to the cartilage surface creating an exposed lubricin layer. The ECM proteins also bind to each other and are shown in the cartilage. Lubricin is also found in the SF.

The binding of collagen II and fibronectin to lubricin was confirmed by solid phase binding assay using collagen II from bovine cartilage and fibronectin from human blood binding to recombinant lubricin fragments. Both proteins were shown to bind to some extent to the mucin domain, however, given the possibility of the involvement of glycosylation in this interaction, further analyses are needed to better understand the recombinant glycosylation compared to normal lubricin to determine if the binding observed is specific. Lubricin bound to fibronectin strongly via the C-terminal fragment (L1078-1404). Lubricin binding to collagen II, on the other hand, is focused to the N-terminal of lubricin and when smaller N-terminal fragments were used could be narrowed to the L105-160 fragment. The unglycosylated N-terminal of lubricin^11^ has been shown to be important for noncovalent and covalent bonding with COMP^28^. In fact, solid phase binding assays between recombinant COMP and the same lubricin fragments used here showed that the lubricin amino acid range of 105 to 202 was responsible for non-covalent binding with COMP, overlapping with the binding regions (L105-160) important for binding to collagen II. The adherence at the termini of lubricin to other cartilage proteins including COMP, collagen II and fibronectin would allow the heavily *O*-glycosylated negatively charged region^11^, suggested to provide the low friction properties of lubricin^12^, to move freely, extending from the articular cartilage surface into the SF (as illustrated in Fig. 6). This extended nature would create a flexible surface that, even with multidirectional movement, would flex to always cover the surface.

In summary, lubricin complexes with COMP, collagen II and fibronectin at the articular cartilage surface, as shown by *in situ* PLA, leaving the lubricin mucin domain exposed at the surface. The COMP-lubricin complexes were also identified deeper into the tissue in this report, suggesting they are produced in the cartilage and migrate to the surface. Although we do not know the binding mechanism involved in these complexes, or how synovial lubricin may also play into this complex formation, it is clear that lubricin is able to bind collagen II as well as fibronectin *in situ* on the cartilage surface. COMP also binds fibronectin^23^ and collagen II^24^, suggesting that a large multi-protein complex may be created at the surface. Understanding the mechanism of the attachment of lubricin to the cartilage surface becomes even more significant as the use of lubricin as a biologic to treat osteoarthritic disease becomes more likely^52^. Overall, it is clear that a multifaceted network is involved to ensure the adherence of lubricin, and as we continue to elucidate the mechanisms involved in retaining boundary lubrication, we become closer to the ability to restore it for the millions of people who need it.

## Methods

### Human SF and cartilage and mouse samples and preparation

Human cartilage biopsies (n = 2), both male aged 67 and 79 years of age, including the subchondral bone were obtained from non-weight bearing ventral areas of the femoral trochlea peripheral to the femoropatellar joint during medial unicompartmental knee arthroplasty surgery with primary, anterior idiopathic OA and intact cruciate ligaments. Biopsies were collected at the Orthopaedic Clinic at Danderyds Hospital (Stockholm, Sweden) and were acquired from the border between macroscopically normal appearing cartilage and areas with obvious cartilage degradation. Biopsies were demineralised until soft enough to section in 175 mM EDTA, 10 mM Tris (pH 6.95), and 3.75% polyvinylpyrrolidone-40 at 4 °C. Enriched lubricin from SF samples was used as control for the MMP assay. SF sample from arthritic patients (n = 1 was collected during aspiration of knee joints at the Rheumatology Clinic, Sahlgrenska University Hospital (Gothenburg, Sweden). All OA and RA patients gave informed consent and all the procedures were approved by the regional ethical review board in Gothenburg (172-15,13/5-2015). All methods were performed in accordance with the relevant guidelines and regulations.

Paw tissue samples from 7 month old COMP deficient 129/sv mice described here as COMP KO mice^32^ and equivalent wild type (WT) mice (n = 2) were used for immunohistochemistry analyses. This work, undertaken at Lund University, Lund, Sweden, was approved by the regional Lund-Malmö laboratory animal ethics committee (M9-08). All methods were performed in accordance with the relevant guidelines and regulations.

### Immunohistochemistry

#### Human biopsy samples

Acetone fixed human cartilage biopsy cryosections were blocked (10% fetal bovine serum, PBS). Negative controls (normal serum or isotype matched negative controls) or primary antibodies (2 µg/ml), mouse anti-human COMP (clone 16F12, Biovendor R&D) and polyclonal rabbit anti-lubricin (P3-118, Thermo Scientific) were incubated separately. Alexa Flour 488 conjugated goat anti-mouse IgG (Jackson ImmunoResearch) and Alexa Flour 594 conjugated goat anti-rabbit IgG (Jackson ImmunoResearch) were diluted 1000 fold and incubated together. Tissue was mounted with Prolong® Gold anti-fade reagent with 4′,6-diamidino-2-phenylindole nuclear stain. Human biopsy tissues were also stained with Haematoxylin and eosin.

#### COMP KO mouse samples

Mouse paw tissue paraffin embedded sections from 7 month old COMP deficient 129/Sv mice^32^ and age matched WT mice were analysed. Paraffin removal and then antigen retrieval were obtained by incubation in an alkaline solution (K8004 EnVision FLEX Target Retrieval Solution, High pH 9.0, DAKO, Denmark A/S, Glostrup, Denmark) at 85 °C for 40 min. Sections were cooled in washing buffer (K8007 EnVision FLEX Wash Buffer, DAKO) and blocked for 1 h at room temperature (RT) with 2% normal donkey serum in PBS (Jackson ImmunoResearch Laboratories, INC., West Grove, PA), also used for antibody dilution. Primary rabbit anti-lubricin antibody (P3-118, Thermo Scientific) at 2 µg/ml was incubated overnight at 4 °C followed by incubation with the secondary antibody for 1 h at RT with 3 × 5 min intermediary washes with washing buffer. The secondary antibody was either Rhodamine Red-X-conjugated, donkey anti-rabbit (711-296-162, Jackson ImmunoResearch, diluted 1 in 1000), or biotin-conjugated, donkey anti-rabbit (Jackson ImmunoResearch, diluted 1 in 1000) visualised after incubation with streptavidin-FITC for 30 min at RT. Negative control was performed with primary antibody omitted. Finally, sections were mounted as above and images were captured in a Zeiss Axioscope 2 Plus fluorescence microscope and images were cropped and contrast adjusted for the complete image in Adobe Photoshop CC.

### Proximity ligation assay (PLA)

Human cartilage biopsy tissue sections were permeabilised with 0.05% Triton in Tris-buffered saline (TBS) (50 mM Tris pH 7.6, 150 mM NaCl) for 15 min and rinsed twice in TBS. Tissue sections for collagen II antibody were also treated with hyaluronidase from bovine testes (H3506, Sigma-Aldrich) at 8000 U/ml in PBS, pH 5.5, for 1 h at 37 °C. Slides were blocked with Duolink Blocking solution (Olink Bioscience) for 45 min at 37 °C incubated in a humidity chamber. Primary antibodies were diluted in Duolink Antibody Diluent (Olink Bioscience) as follows: mouse anti-COMP (clone 16F12, Biovendor Research and Diagnostic Products) at 1 µg/ml, rabbit anti-lubricin (PA3-118, Thermo Scientific) at 1 µg/ml, mouse anti-lubricin (clone 5C11, Millipore MABT400) at 1 µg/ml, goat anti-collagen II α1 (COL2A1 (C-19), sc-7763, Santa Cruz Biotechnology) at 1 µg/ml, rabbit anti-fibronectin (ab32419, abcam) at 0.06 µg/ml, and incubated overnight at 4 °C. The slides were washed in TBS with 0.05% Tween-20 (TBS-T) for 3 × 5 min with gentle agitation as for all following washes. Secondary probes (Olink Bioscience) were diluted 1:5 in Duoink Antibody Diluent (Olink Bioscience) and incubated in a humidity chamber for 90 min at 37 °C. The slides were washed and ligation solution (Olink Bioscience) was added for 30 min at 37 °C, followed by washing and incubation with amplification solution for 90 min at 37 °C. Finally, the slides were washed with Buffer- B (Olink Bioscience) and counterstained with Hoechst (Life technologies) and FITC-conjugated phalloidin (Sigma) at RT for 10 min. Appropriate technical controls with primary antibody omission were included to test background levels. Images were captured using a Zeiss Imager Z2 microscope with AxioCam MRm Rev.3 camera and Zen pro 2011 software.

### Solid phase binding assays

Recombinant lubricin fragments were produced and characterised as described previously^28^. FLAG-tagged recombinant human lubricin with a truncated mucin-like domain (without AA 403–870) was produced in 293 F cells using p3xFLAG-CMV-8 vector (Sigma-Aldrich) and purified on anti-FLAG beads. The same method was used to produce four lubricin fragments named L25-221 (all molecular weights are calculated, MW 21.9 kDa), L220-402 (MW 19.2 kDa), L871-1078 (MW 22.6 kDa), L1079-1404 (MW 37.2 kDa). GST-tagged fragments of the *N*-terminal domain divided by exon boundaries (exons 2-5), named L25-72 (MW 5.3 kDa), L67-109 (MW 5.0 kDa), L105-160 (MW 6.0 kDa), L155-202 (MW 5.2 kDa), were produced using pGEX-5X-3 vector (GE Healthcare) and Rosetta 2 *E. coli* (Novagen), purified in native conditions using glutathione beads (Pierce). Lubricin fragments have been recently characterised^28^. Fibronectin was purified from human blood^53^ and collagen II was extracted from bovine cartilage^54^ as previously described.

Fibronectin or collagen II (2 µg/ml in PBS) were coated overnight on a 96-well plate, rinsed twice with TBS and blocked for 1 h with 5% BSA in TBS. After a TBS rinse, the plates were incubated with lubricin fragments at 2 µg/ml in wash buffer (TBS + 0.1% BSA + 0.1% Tween-20) or BSA as a negative control, for 2 h. Plates were then washed three times with wash buffer and incubated with mouse anti-FLAG (Genscript) or rabbit anti-GST (Abcam) antibodies at 1 µg/ml in wash buffer for 1 h. The plates were then incubated with anti-mouse or anti-rabbit HRP-conjugated antibodies (DAKO) at 0.2 µg/ml in wash buffer for 1 h. After washing, binding was detected using TMB substrate (ThermoFisher), signal stopped with sulphuric acid and absorbance read at 450 nm. Assays were performed in triplicate and repeated twice, error bars are standard deviation. Data was analyses by one-way ANOVA followed by Bonferroni’s Multiple Comparison Test to compare each of the fragments with the BSA standard using GraphPad Prism version 5.

### MMP assay and Western blot analysis

#### MMP assay

Human MMP-9 protease was obtained as proenzymes (R&D systems, Inc.) and activated with 1 mM ρ-aminophenylmercuric acetate at 37 °C in reaction buffer (50 mM Tris, 10 mM CaCl_2_, 150 mM NaCl, 0.05% Brij 35, pH 7.5) according to the manufacturer’s instruction. Four pieces of cartilage (20–25 mg) were removed from intact biopsy specimens after softening for sectioning. Cartilage pieces were rinsed with reaction buffer before the addition of 300 µl of reaction buffer and 1 µg of activated or pro-enzyme. Aliquots of each supernatant were removed at 1, 2 and 4 h. Enriched synovial lubricin was tested under the same conditions to evaluate its susceptibility to MMP-9.

#### Western blotting analysis

MMP assay samples were separated by 3–8% Tris/acetate gels. Gels were transferred to PVDF membrane, blocked (3% bovine serum albumin in PBS), and probed with mouse anti-human lubricin (mAb 13, Pfizer Research) followed by HRP conjugated rabbit anti-mouse immunoglobulins (DakoCytomation). Images are shown without alteration and complete Western blots shown in supplementary information Fig. S1.

### Data availability

All data generated or analysed during this study are included in this published article and its supplementary information.

## Electronic supplementary material

Supplementary Informantion

## References

[1] Chen D (2017). Osteoarthritis: toward a comprehensive understanding of pathological mechanism. Bone research.

[2] Lee AS (2013). A current review of molecular mechanisms regarding osteoarthritis and pain. Gene.

[3] Smolen JS, Aletaha D, McInnes IB (2016). Rheumatoid arthritis. Lancet.

[4] Rosman Z, Shoenfeld Y, Zandman-Goddard G (2013). Biologic therapy for autoimmune diseases: an update. BMC medicine.

[5] Allen KD (2016). *Osteoarthritis: Models for appropriate care across* the disease continuum. Best practice & research. Clinical rheumatology.

[6] Flannery CR (1999). Articular cartilage superficial zone protein (SZP) is homologous to megakaryocyte stimulating factor precursor and Is a multifunctional proteoglycan with potential growth-promoting, cytoprotective, and lubricating properties in cartilage metabolism. Biochem Biophys Res Commun.

[7] Gleghorn JP, Bonassar LJ (2008). Lubrication mode analysis of articular cartilage using Stribeck surfaces. Journal of biomechanics.

[8] Jones AR (2007). Binding and localization of recombinant lubricin to articular cartilage surfaces. Journal of orthopaedic research: official publication of the Orthopaedic Research Society.

[9] Nugent-Derfus GE, Chan AH, Schumacher BL, Sah RL (2007). PRG4 exchange between the articular cartilage surface and synovial fluid. Journal of orthopaedic research: official publication of the Orthopaedic Research Society.

[10] Estrella RP, Whitelock JM, Packer NH, Karlsson NG (2010). The glycosylation of human synovial lubricin: implications for its role in inflammation. Biochem J.

[11] Ali L (2014). The O-glycomap of lubricin, a novel mucin responsible for joint lubrication, identified by site-specific glycopeptide analysis. Mol Cell Proteomics.

[12] Jay GD, Hong BS (1992). Characterization of a bovine synovial fluid lubricating factor. II. Comparison with purified ocular and salivary mucin. Connective tissue research.

[13] Lee DW, Banquy X, Israelachvili JN (2013). Stick-slip friction and wear of articular joints. Proc Natl Acad Sci USA.

[14] Jay GD, Waller KA (2014). The biology of lubricin: near frictionless joint motion. Matrix Biol.

[15] Camarero-Espinosa, S., Rothen-Rutishauser, B., Foster, E. J. & Weder, C. Articular cartilage: from formation to tissue engineering. *Biomaterials science*, 10.1039/c6bm00068a (2016). 10.1039/c6bm00068a 26923076

[16] Theocharis AD, Skandalis SS, Gialeli C, Karamanos NK (2016). Extracellular matrix structure. Advanced drug delivery reviews.

[17] Eyre DR (1991). The collagens of articular cartilage. Seminars in arthritis and rheumatism.

[18] Graham HK, Holmes DF, Watson RB, Kadler KE (2000). Identification of collagen fibril fusion during vertebrate tendon morphogenesis. The process relies on unipolar fibrils and is regulated by collagen-proteoglycan interaction. J Mol Biol.

[19] Potts JR, Campbell ID (1994). Fibronectin structure and assembly. Current opinion in cell biology.

[20] Kadler KE, Hill A, Canty-Laird EG (2008). Collagen fibrillogenesis: fibronectin, integrins, and minor collagens as organizers and nucleators. Current opinion in cell biology.

[21] Oldberg A, Antonsson P, Lindblom K, Heinegard D (1992). COMP (cartilage oligomeric matrix protein) is structurally related to the thrombospondins. J Biol Chem.

[22] Chen FH (2007). Interaction of cartilage oligomeric matrix protein/thrombospondin 5 with aggrecan. J Biol Chem.

[23] Di Cesare PE (2002). Matrix-matrix interaction of cartilage oligomeric matrix protein and fibronectin. Matrix Biol.

[24] Chang DP, Guilak F, Jay GD, Zauscher S (2014). Interaction of lubricin with type II collagen surfaces: adsorption, friction, and normal forces. Journal of biomechanics.

[25] Holden P (2001). Cartilage oligomeric matrix protein interacts with type IX collagen, and disruptions to these interactions identify a pathogenetic mechanism in a bone dysplasia family. J Biol Chem.

[26] Rosenberg K, Olsson H, Morgelin M, Heinegard D (1998). Cartilage oligomeric matrix protein shows high affinity zinc-dependent interaction with triple helical collagen. J Biol Chem.

[27] Vranka J (2001). Selective intracellular retention of extracellular matrix proteins and chaperones associated with pseudoachondroplasia. Matrix Biol.

[28] Flowers, S. A. *et al*. Cartilage oligomeric matrix protein forms protein complexes with synovial lubricin via non-covalent and covalent interactions. *Osteoarthritis and Cartilage* in press, 10.1016/j.joca.2017.03.016 (2017). 10.1016/j.joca.2017.03.016 28373131

[29] Andresen Eguiluz RC (2015). Fibronectin mediates enhanced wear protection of lubricin during shear. Biomacromolecules.

[30] Reesink HL (2016). Galectin-3 Binds to Lubricin and Reinforces the Lubricating Boundary Layer of Articular Cartilage. Scientific reports.

[31] Bonnevie ED, Puetzer JL, Bonassar LJ (2014). Enhanced boundary lubrication properties of engineered menisci by lubricin localization with insulin-like growth factor I treatment. Journal of biomechanics.

[32] Svensson L (2002). Cartilage oligomeric matrix protein-deficient mice have normal skeletal development. Molecular and cellular biology.

[33] Soderberg O (2006). Direct observation of individual endogenous protein complexes *in situ* by proximity ligation. Nature methods.

[34] Nagase, H. In *Matrix Metalloproteinase inhibitors in cancer therapy* (eds N.J. Clendeninn & K. Appelt) Ch. 2, (Humana Press, 2001).

[35] Ganu V (1998). Inhibition of interleukin-1alpha-induced cartilage oligomeric matrix protein degradation in bovine articular cartilage by matrix metalloproteinase inhibitors: potential role for matrix metalloproteinases in the generation of cartilage oligomeric matrix protein fragments in arthritic synovial fluid. Arthritis and rheumatism.

[36] Muller G, Michel A, Altenburg E (1998). COMP (cartilage oligomeric matrix protein) is synthesized in ligament, tendon, meniscus, and articular cartilage. Connective tissue research.

[37] Berenbaum F (2013). Osteoarthritis as an inflammatory disease (osteoarthritis is not osteoarthrosis!). Osteoarthritis and cartilage / OARS, Osteoarthritis Research Society.

[38] Jones AR (2009). Modulation of lubricin biosynthesis and tissue surface properties following cartilage mechanical injury. Arthritis and rheumatism.

[39] Di Cesare PE (1996). Increased degradation and altered tissue distribution of cartilage oligomeric matrix protein in human rheumatoid and osteoarthritic cartilage. J Orthop Res.

[40] DiCesare P, Hauser N, Lehman D, Pasumarti S, Paulsson M (1994). Cartilage oligomeric matrix protein (COMP) is an abundant component of tendon. FEBS Lett.

[41] Smith RK, Zunino L, Webbon PM, Heinegard D (1997). The distribution of cartilage oligomeric matrix protein (COMP) in tendon and its variation with tendon site, age and load. Matrix Biol.

[42] Anthony S, Munk R, Skakun W, Masini M (2015). Multiple epiphyseal dysplasia. The Journal of the American Academy of Orthopaedic Surgeons.

[43] Posey KL, Alcorn JL, Hecht JT (2014). Pseudoachondroplasia/COMP - translating from the bench to the bedside. Matrix Biol.

[44] Posey KL (2009). An inducible cartilage oligomeric matrix protein mouse model recapitulates human pseudoachondroplasia phenotype. The American journal of pathology.

[45] Gamble C, Nguyen J, Hashmi SS, Hecht JT (2015). Pseudoachondroplasia and painful sequelae. American journal of medical genetics. Part A.

[46] Briggs MD (1995). Pseudoachondroplasia and multiple epiphyseal dysplasia due to mutations in the cartilage oligomeric matrix protein gene. Nat Genet.

[47] Hecht JT (1995). Mutations in exon 17B of cartilage oligomeric matrix protein (COMP) cause pseudoachondroplasia. Nat Genet.

[48] Hecht JT (2004). Chondrocyte cell death and intracellular distribution of COMP and type IX collagen in the pseudoachondroplasia growth plate. Journal of orthopaedic research: official publication of the Orthopaedic Research Society.

[49] Jones KL, Brown M, Ali SY, Brown RA (1987). An immunohistochemical study of fibronectin in human osteoarthritic and disease free articular cartilage. Annals of the rheumatic diseases.

[50] Carnemolla B (1984). Characterization of synovial fluid fibronectin from patients with rheumatic inflammatory diseases and healthy subjects. Arthritis and rheumatism.

[51] Majd SE (2014). Both hyaluronan and collagen type II keep proteoglycan 4 (lubricin) at the cartilage surface in a condition that provides low friction during boundary lubrication. Langmuir: the ACS journal of surfaces and colloids.

[52] Waller, K. A. *et al*. Intra-articular Recombinant Human Proteoglycan 4 Mitigates Cartilage Damage After Destabilization of the Medial Meniscus in the Yucatan Minipig. *The American journal of sports medicine*, 363546516686965, 10.1177/0363546516686965 (2017). 10.1177/0363546516686965 PMC5453820 28129516

[53] Retta SF, Ferraris P, Tarone G (1999). Purification of fibronectin from human plasma. Methods Mol Biol.

[54] Vogel KG, Paulsson M, Heinegard D (1984). Specific inhibition of type I and type II collagen fibrillogenesis by the small proteoglycan of tendon. Biochem J.

