# Supplementary material for: Lubricin binds cartilage proteins, cartilage oligomeric matrix protein, fibronectin and collagen II at the cartilage surface
Source: Sci Rep. 2017 Oct 13;7:13149. doi: 10.1038/s41598-017-13558-y (PMC5640667; doi:10.1038/s41598-017-13558-y)

SUPPLEMENTARY INFORMATION

Lubricin binds cartilage proteins, cartilage oligomeric matrix protein, fibronectin and collagen II at the cartilage surface

Sarah A Flowers, Agata Zieba, Jessica Örnros, Chunsheng Jin, Ola Rolfson, Lena I Björkman, Thomas Eisler, Sebastian Kalamajski, Masood Kamali-Moghaddam, and Niclas G Karlsson

Figure S1. Complete Western blot images of Figure 5. (A) Complete Western blot of cartilage biopsy tissue digested over a time course with activated and pro-MMP-9. This image was cropped and is shown in Fig 5A in manuscript. Std is purified lubricin. (B) Complete Western blot of purified lubricin digested over a time course with activated MMP-9. This image was cropped and is shown in Fig 5B in manuscript.

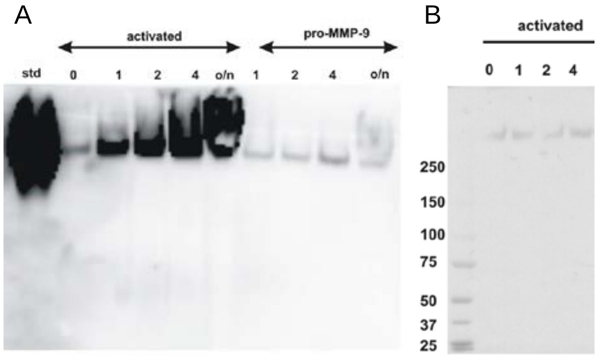

Supplement: Supplementary file 1 — Supplementary Informantion [file 41598_2017_13558_MOESM1_ESM.pdf]
